# Trends of blood-borne infectious diseases in a rural blood donation center of southeast Gabon (Koula-Moutou)

**DOI:** 10.11604/pamj.2018.31.81.16331

**Published:** 2018-10-03

**Authors:** Cyrille Bisseye, Landry-Erik Mombo, Stéphane Meyet Me Bie, Apollinaire Edou, Jean Marie Eko-Mba, Jean-Charles Etho-Mengue, Kévin Mbacky, Arnaud Mongo-Delis, Bertrand M’batchi, Bolni Marius Nagalo

**Affiliations:** 1 Laboratoire de Biologie Moléculaire et Cellulaire (LABMC), Université des Sciences et Techniques de Masuku, BP 943, Franceville, Gabon; 2 Centre Hospitalier Régional Paul Moukambi (CHRPM), BP 03 Koula-Moutou, Gabon; 3 Centre de Traitement ambulatoire de Koula-Moutou, BP 383 Koula-Moutou, Gabon; 4 Division of Hematology and Oncology, Mayo Clinic, 13400 E, Shea Blvd Scottsdale, 85259 AZ, USA

**Keywords:** Seroprevalence, blood-borne pathogens, trends, Koula-Moutou, Gabon

## Abstract

**Introduction:**

Blood-borne pathogens such as human immunodeficiency virus (HIV), hepatitis B and C (HBV and HCV) viruses and *Treponema pallidum* remain a major public health problem in sub-Saharan Africa. The purpose of this study was to assess the frequency and clinical implications of HIV, HBV, HCV and *Treponema pallidum* markers in blood donors in a rural area of Southeast Gabon (Koula-Moutou) from 2012 to 2017.

**Methods:**

Hepatitis B surface antigen (HBsAg), anti-HIV, anti-HCV and anti-*Treponema pallidum* antibodies were screened using rapid diagnostic tests (RDTs).

**Results:**

Of a total of 5,706 blood donors, 1,054 (18.5%) were seropositive for at least one infectious marker and 59 (5.6%) had serologic evidence of multiple infections. The overall seroprevalence of HIV, HBsAg, HCV, and syphilis was 3.1%; 5.9%; 6.2% and 3.3%, respectively. HIV, syphilis and HCV distributions were associated with neither the sex nor the age of the donors. Only HBsAg seroprevalence was significantly higher in donors of the age group 26-35 years old compared to donors of the age group 36-45 years (OR = 1.43 (95% CI: 1.01-2.04), P = 0.045). There was a significant increase in the frequencies of HIV and syphilis and a regression of HBsAg and HCV among blood donors.

**Conclusion:**

This study presents the epidemiology of the main pathogens detected in blood donors in a rural area in Gabon. We found that the overall distribution of transfusion transmitted infectious diseases were lower than those observed in the general population but could be underestimated due to the use of RDTs in the screening process of the blood donations.

## Introduction

Blood transfusion remains a challenging medical procedure in Sub-Saharan Africa (SSA) due to the high prevalence of infectious agents [1]. Several factors affect the safety of blood transfusion in Gabon. These include chronic shortage of blood, high number of first-time donors, lack of qualified personnel and financial resources as well as elsewhere else in the continent [2]. While blood utilization is rising in rural areas due to the high incidence of infectious diseases such as malaria, typhoid fever, the screening of pathogenic agents is still performed using RDTs. Comparatively, in the big cities like Libreville, high-performance equipment are available, thus guaranteed a much safer blood safety environment. In a recent report, a prevalence of 7.28% for HBsAg was reported among first-time donors in Libreville, whilst a 4.1%, 4.9% were reported in the general population for HIV and HCV infections respectively [3-5]. In a previous study conducted in Koula-Moutou, a rural area, the seroprevalences of 1.3%, 3.3%, 4.9% and 1.6% for HIV, HBV, HCV and syphilis were reported, respectively [6]. Given that little is known on the epidemiology of transfusion transmitted infections (TTIs) in rural areas of Gabon. We have set for this study the goal of assessing the distribution of HIV, HBsAg, HCV and syphilis serological markers among blood donors in a rural area (Koula-Moutou). This epidemiological data will serve as a springboard to advocate for a better screening of TTIs using more robust methods such as 4^th^ generation Enzyme-Linked Immunosorbent Assay (ELISA) and Nucleic acid testing's (NATs) which will improve local population heath.

## Methods

**Study population:** A retrospective analysis of blood donor data from 2012 to 2017 was carried out at the Centre Hospitalier regional Paul Moukambi (CHRPM). Individuals aged 17 to 65 with a weight >50 kg were eligible for blood donations after completing a questionnaire to exclude previously transfused persons, pregnant women, people with signs of hepatitis or signs of any other infection, and those with risky sexual behavior in the three months prior to blood donation at CHRPM. All blood donations were tested for HIV, Hepatitis B surface (HBsAg), HCV and syphilis. The socio-demographic characteristics of the blood donors were recorded in a database and venous blood was collected in the blood bags following the standard procedure.

**Determination of blood and rhesus groups:** ABO and rhesus blood groups were determined using anti-A, anti-B, anti-AB and anti-D antisera (Cypress diagnosis, Belgium) according to the manufacturer's recommendations.

**Serological markers detection:** Screening for all infections in blood donors was done by rapid diagnostic tests (RDTs). Anti-HIV-1 & 2 antibodies were detected by DETERMINE (Abbott, USA) and SD Bioline HIV-1/2 3.0 (Standard Diagnostics, INC, Gyeonggy-do, South Korea). In blood donors with discordant HIV serology after both tests, the ImmunocombII HIV-1 & 2 Bispot test (Orgenics, Yavne, Israel) was used for confirmation. Hepatitis B surface antigen (HBsAg) and anti-HCV antibodies were detected by the Determine ™ HBV and ImmunocombII HCV kit, (Alere S.A.S. Jouy En Josas, France) according to the manufacturer's recommendations. Anti-*Treponema pallidum* antibodies have been detected by the non-treponemal test RPR (Rapid Plasma Reagin) (BIOLABO, Maizy, France). All RPR-reactive donors were confirmed by the Treponema pallidum Hemagglutination Assay (TPHA) test (Biolabo, Maizy, France).

**Statistical analysis:** Data analysis was done by using the Statistical Package for the Social Sciences (SPSS version 20.0) and EPI-Info version 6.04dfr (CDC, Atlanta, USA). Odds ratios (OR) and 95% confidence intervals are presented. The results were considered significant for P < 0.05.

**Ethical considerations:** This study received the approval of the institutional review board of the Centre Hospitalier Régional Paul Moukambi (CHRPM).

## Results

**Sociodemographic characteristics of Koula-Moutou's blood donors:** The study involved 5,706 blood donors from 2012 to 2017. The majority of donors were male (83.5%). The age of the donors ranged from 17 to 65 years old. The age groups 17-25 years and 26-35 years were the most represented among blood donors with 42.0% and 38.0%, respectively. The majority of blood donors were O blood group (56.4%) and Rh positive (97.3%). Rhesus negative blood donors were underrepresented with 2.7%. All of the blood donors were family/replacement donors (FRD) whereas no voluntary non-remunerated donors (VNRD) were reported. The overall sociodemographic characteristics of blood donors are summarized in Table 1.

**Table 1:** Sociodemographic characteristics of blood donors in Koula-Moutou from 2012 to 2017

	2012	2013	2014	2015	2016	2017	Total
	771(13.5)	864(15.1)	1292(22.6)	546(9.6)	1205(21.1)	1028(18.0)	**5706**
**Sex**							
Male	555(72.0)	651(75.3)	1036(80.2)	478(87.5)	1105(91.7)	940(8.6)	4765(83.5)
Female	216(28.0)	213(24.7)	256(19.8)	68(12.5)	100(8.3)	88(8.6)	941(16.5)
**Age groups**							
17-25 yrs	287(37.2)	359(41.6)	568(44.0)	231(42.3)	522(43.3)	429(41.7)	2396(42.0)
26-35 yrs	287(37.2)	328(38.0)	465(36.0)	214(39.2)	471(39.1)	404(39.3)	2169(38.0)
36-45 yrs	176(22.8)	158(18.3)	232(18.0)	94(17.2)	200(16.6)	170(16.5)	1030 (18.1)
˃45 yrs	21(2.7)	19(2.2)	27(2.1)	7 (1.3)	12(1.0)	25(2.4)	111(1.9)
**Type of donors**							
FRD	771 (100)	864 (100)	1292(100)	546(100)	1205(100)	1028(100)	5706(100)
VNRD	0(0)	0(0)	0(0)	0(0)	0(0)	0(0)	0(0)
**Blood Group**							
O	424(55.0)	502(58.1)	717(55.5)	298(54.6)	692(57.4)	587(57.1)	3220(56.4)
A	179(23.2)	214(24.8)	319(24.7)	118(21.6)	279(23.2)	258(25.1)	1367(24.0)
B	147(19.1)	133(15.4)	228(17.6)	119(21.8)	224(18.6)	172(16.7)	1023(17.9)
AB	21(2.7)	15(1.7)	28(2.2)	11(2.0)	10(0.8)	11(1.1)	96(1.7)
**Rhesus**							
Positive	754(97.8)	839(97.1)	1270(98.3)	527(96.5)	1164(96.6)	997(97.0)	5551(97.3)
Negative	17(2.2)	25(2.9)	22(1.7)	19(3.5)	41(3.4)	31(3.0)	155(2.7)

**Figure 1:**
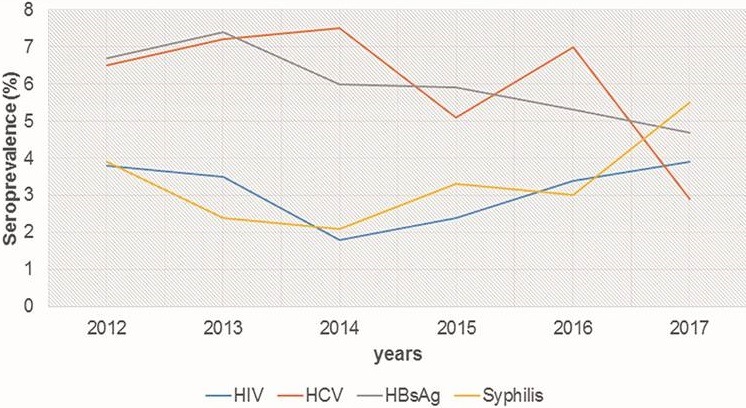
Annual variation of infectious markers among blood donors at Koula-Moutou from 2012 to 2017

**Seroprevalence and trends of HIV, HBV, HCV and Syphilis in blood donors from 2012 to 2017:** Of the 5,706 blood donors 18.5% (1,054/5,706) were positive for at least one infectious marker. The overall seroprevalence of HIV, HBsAg, HCV and Syphilis was 3.1%; 5.9%; 6.2% and 3.3% among blood donors in Koula-Moutou. Seroprevalence of HIV and syphilis increased significantly among donors between 2014 and 2017 (p = 0.028, p = 0.001). The lowest seroprevalence of these pathogens were observed in 2014 (1.8% and 2.1%), while the highest seroprevalence of 3.9% and 5.5% were obtained in 2017 (Table 2 and Figure 1). Overall there was a decrease in HBsAg seroprevalence in donors from Koula-Moutou from 2012 to 2017. However, this regression was not statistically significant (p = 0.151). The seroprevalence of HCV has shown variable fluctuation. It increased between 2012 and 2014 from 6.5% to 7.5%. Then fell to 5.1% in 2015 before rising to 7.0% in 2016 and finally dropping sharply to 2.9% in 2017 (Table 2 and Figure 1).

**Table 2:** Annual seroprevalence of HIV, HBV, HCV and syphilis among Koula-Moutou’s blood donors from 2012 to 2017

Year	Total	VIH +N(%)	VHC + N(%)	VHB + N(%)	Syphilis + N(%)
2012	771	29(3.8)	50(6.5)	52(6.7)	30(3.9)
2013	864	30(3.5)	62(7.2)	64(7.4)	21(2.4)
2014	1292	23(1.8)	97(7.5)	78(6.0)	27(2.1)
2015	546	13(2.4)	28(5.1)	32(5.9)	18(3.3)
2016	1205	41(3.4)	84(7.0)	64(5.3)	36(3.0)
2017	1028	40(3.9)	30(2.9)	48(4.7)	57(5.5)
Total	5706	176(3.1)	351(6.2)	338(5.9)	189(3.3)
P-value	-	0.028	0.001	0.151	0.001

**Socio-demographic characteristics of blood donors and HIV and HBsAg seroprevalence:** Seroprevalence of HIV was 3.1% in blood donors. It was two-fold higher in 2016 and 2017 compared to 2014 (p = 0.015 and p = 0.003). It was not associated with sex or age of blood donors, although the age group 36-45 years with 2.9% was the least infected (Table 3). The overall seroprevalence of HBsAg was 5.9% (338/5706). HBV seropositive donors were significantly 1.5 times higher in 2013 than in 2017. No difference was observed for HBV infection by comparing the other years to 2017. HBV infection was 6.2% in men and 4.7% among women, but the observed difference was not statistically significant (Table 3). HBsAg seroprevalence was significantly higher in donors of the age group 26-35 years old compared to donors of the age group 36-45 years (OR = 1.43 (95% CI: 1.01-2.04), P = 0.045) (Table 3).

**Table 3:** Seroprevalence of HIV and HBsAg infection by sociodemographic characteristics of blood donors in Koula-Moutou

	HIV				HBsAg		
	Total	N (%)	OR (95% CI)	P-value	N (%)	OR (95% CI)	P-value
**Year of donation**							
2012	771	29 (3.8)	2.16 (1.20-3.89)	0.008	52 (6.7)	1.48 (0.97-2.25)	0.072
2013	864	30 (3.5)	1.98 (1.11-3.56)	0.019	64 (7.4)	1.63 (1.09-2.45)	0.016
2014	1292	23 (1.8)	Reference	Reference	78 (6.0)	1.31 (0.89-1.93)	0.176
2015	546	13 (2.4)	1.35 (0.64-2.80)	0.506	32 (5.9)	1.27 (0.78-2.06)	0.366
2016	1205	41 (3.4)	1.94 (1.13-3.36)	0.015	64 (5.3)	1.15 (0.77-1.71)	0.555
2017	1028	40 (3.9)	2.23 (1.29-3.88)	0.003	48 (4.7)	Reference	Reference
**Sex**							
Male	4765	146(3.1)	Reference	Reference	294 (6.2)	1.34 (0.96-1.88)	0.089
Female	941	30 (3.2)	1.07 (0.71-1.63)	0.803	44 (4.7)	Reference	Reference
**Age groups**							
17-25 yrs	2396	76 (3.6)	1.09 (0.70-1.72)	0.768	145 (6.1)	1.35 (0.95-1.92)	0.098
26-35 yrs	2169	66 (3.0)	1.05 (0.66-1.66)	0.928	139 (6.4)	1.43 (1.01-2.04)	0.045
36- 45 yrs	1030	30 (2.9)	Reference	Reference	47 (4.6)	Reference	Reference
˃ 45 yrs	111	4 (3.6)	1.25 (0.37-3.80)	0.565	7 (6.3)	1.41 (0.57-3.34)	0.558

**Sociodemographic characteristics of blood donors and seroprevalence of HCV and syphilis:** The overall seroprevalence of HCV was 6.2% (351/5706). This seroprevalence has varied annually. It was almost two-and-a-half fold higher in 2012, 2013, 2014, and 2016 than in 2017. HCV seropositivity was almost equal in women (6.3%) and men (6.1%) and was not associated with the age of blood donors (Table 4). The overall seroprevalence of Syphilis was 3.3% (189/5706). This seroprevalence has varied annually between 2012 and 2017. It was two to three higher in 2012 and 2017 than in 2014. It was 3.4% for men and 2.9% for women. But the difference observed was not significant (p = 0.465). Syphilis was not associated with the age of blood donors even It was higher in blood donors > 45 years (Table 4).

**Table 4:** Seroprevalence of HCV and syphilis infection by sociodemographic characteristics of Koula-Moutou’s Blood Donors

	HCV				Syphilis		
	Total	N (%)	OR (95% IC)	P-value	N (%)	OR (95% IC)	P-value
**Year of donation**							
2012	771	50(6.5)	2.31 (1.42-3.76)	< 0.001	30(3.9)	1.90 (1.09-3.32)	0.023
2013	864	62(7.2)	2.57 (1.61-4.12)	< 0.001	21(2.4)	1.17 (0.63-2.15)	0.706
2014	1292	97(7.5)	2.70 (1.75-4.19)	< 0.001	27(2.1)	Reference	Reference
2015	546	28(5.1)	1.80 (1.03-3.14)	0.038	18(3.3)	1.60 (0.84-3.03)	0.172
2016	1205	84(7.0)	2.49 (1.60-3.90)	< 0.001	36(3.0)	1.44 (0.85-2.46)	0.192
2017	1028	30(2.9)	Reference	Reference	57(5.5)	2.75 (1.69-4.50)	<0.001
Sex							
Male	4765	292 (6.1)	Reference	Reference	162 (3.4)	1.19 (0.78-1.84)	0.465
Female	941	59 (6.3)	1.02 (0.76-1.38)	0.927	27 (2.9)	Reference	Reference
Age groups							
17-25 yrs	2396	146 (6.1)	1.07 (0.83-1.38)	0.634	74 (3.1)	1.02 (0.72-1.44)	0.997
26-35 yrs	2169	124 (5.7)	Reference	Reference	66 (3.0)	Reference	Reference
36- 45 yrs	1030	73 (7.1)	1.26 (0.92-1.71)	0.153	43 (4.2)	1.39 (0.92-2.09)	0.122
˃ 45 yrs	111	8 (7.2)	1.28 (0.56-2.79)	0.655	6 (5.4)	1.82 (0.69-4.49)	0.161

## Discussion

A significance differences exist in the frequencies of TTIs in blood donation between urban areas and rural areas in SSA which is mainly due to the profiles of the donors. In Gabon, the majority of blood donors in Koula-Moutou, a rural area, are FRD, whereas in urbans areas the number of VNRD predominates [6]. As expected in this study, we also found that all blood donations were collected from FRDs. This finding is also consistent with earlier studies that showed a predominance of family donors in SSA, reaching 75-80% of the total blood donors [2]. This predominance could be explained by many sociocultural factors which are still strong in rural areas. Indeed, relatives, friends, neighbors and peers are important support for patients; therefore provide a direct source of blood collection. In addition of being more accessible, donations from FRD are inexpensive. In contrast, recruiting VNRD requires an establishment of a strong and often costly awareness and recruitment programs which are difficult to maintain because of the limited financial resources in blood centers [7, 8]. The majority of blood donors were men (83.5%) compared to women (16.5%). This percentage of men is similar to those found in studies across Africa [9, 10]. Diverse medical contraindications might prevent women of donating blood including pregnancy, menstruation, breastfeeding, anemia and some cultural beliefs [9, 11-13]. The 17-35 age group (80.0%) was the most represented among blood donors. This high proportion of young blood donors has been found in most African countries, and this can be explained by the younger demographic structure of the African population [14]. In this study, 18.5% of blood donors were seropositive for at least one infectious marker. The overall seroprevalence of transfusion-transmitted infection (TTI) in Koula-Moutou blood donors is higher than that of 11.5% reported in a previous study in Ethiopia [15]. However, it is lower than TTIs seroprevalence of 21.2% and 19.3% respectively observed in Cameroon [11] and Nigeria [10].

These suggest that there is a geographical difference on the epidemiology of the TTIs in SSA. The overall seroprevalence of HIV was 3.1% in blood donors. We found in this study a significant annual variation among blood donors between 2014 and 2017, increasing from 1.8% to 3.9%. This increase in HIV seroprevalence among blood donors is at odds with the 50% reduction of new HIV infections in the general population since 2010 in Gabon but remains similar to the national seroprevalence of 4.1% [4]. The seroprevalence of HIV reported in this study is higher than the seroprevalence of 2.5% and 2.21% observed respectively in Cameroon [11] and in Burkina Faso [16]. This seroprevalence is similar to the 3.1% and 3.8% seroprevalence reported respectively in Nigeria [17] and in Ethiopia [9]. Higher HIV seroprevalence of 8.5% and 7.83% were observed, respectively, in blood donors in Mozambique [18] and Equatorial Guinea [19]. This discrepancy might indicate a difference in the performances of the type of diagnostic tests used in the screening of blood donations in the different studies: RDT (in our study) versus 3^rd^ and 4^th^ generation ELISA. Previous investigations have shown the low sensitivity and specificity of RDTs compared to third and fourth-generation immunoenzymatic assays in the diagnosis of HIV in blood donors in SSA [20-22]. Neither sex nor ages were significantly associated with HIV infection in blood donors. Our findings contrasted with those reported by Nagalo *et al* [16] who showed higher seroprevalence of HIV in blood donors aged over 40 in Koudougou, a semi-urban area of Burkina Faso. The overall seroprevalence of HBsAg in donors was 5.9% which showed a non-significant annual decrease from 6.7% in 20^12^ to 4.7% in 2017. The seroprevalence of HBsAg of 5.9% found in this study is higher than those of 3.3% reported among Koula-Moutou blood donors in a previous study [6] and the 3.8% reported in Cameroon [23]. Seroprevalence of HBsAg was lower than that of 7.28% reported recently in a study in Libreville blood donors [3]. However, higher seroprevalences of HBsAg of 10.01%; 14.96%; 18.6% and 22.3% were reported respectively in Equatorial Guinea [19], Burkina Faso [16], Nigeria [17] and Ethiopia [24]. Seroprevalence of HBsAg was not significantly associated with donor sex. Our results don't correlate with the studies conducted in Namibia [12] and Gabon [3] which reported a significantly high seroprevalence of HBsAg in male blood donors. Seroprevalence of HBV was significantly higher in blood donors of the age group 26-35 years which is consistent with a recent report in Gabon [3].

The overall seroprevalence of HCV in blood donors was 6.2%. It has seen annual variations, falling significantly between 2012 and 2017 from 6.5% to 2.9%. The HCV seroprevalence of 6.2% found in this study is higher than the seroprevalences of 1.1% and 1.2% reported in previous studies in Nigeria [17], Libya and Niger, respectively [25, 26]. However, it is similar to that of 6.04% reported in Gabon [27]. No significant differences were found in comparing the seroprevalence of HCV between men and women, and between different age groups. The seroprevalence of syphilis was 3.3% with significant annual variations, increasing from 3.9% to 5.5% between 2012 and 2017. This increase is in contradiction with the decline of syphilis from 5.5% to 1.1% observed by WHO in Central Africa [28]. The overall syphilis seroprevalence of 3.3% is higher than the seroprevalences of 0.1%, 1.1% and 1.5% reported respectively in Ethiopia [15], Nigeria [17] and Burkina Faso [29]. However, it is lower than the prevalences 3.96% and 8.1% respectively observed by Nagalo *et al* [16] and Eboumbou Moukoko *et al* [23] in Burkina Faso and Cameroon. The higher seroprevalences of syphilis of 21.51% and 12.7% were observed in Equatorial Guinea [19] and in Tanzania [30]. These variations in the seroprevalences of syphilis can be due on one hand, to the differences of sensitivity and specificity of the diagnostic tests used, and on the other hand to sexual behaviors, accessibility to health care, matrimonial practices, sample size during surveys and donor selection criteria [31]. The seroprevalence of syphilis was not associated with the sex and age of the blood donors. However, it was higher in the age group > 45 years. These results do not support those reported in Nigeria that showed that the most affected age group was 18 to 24 years of age [17]. The higher syphilis seroprevalence found in the age group >45 years could be probably due to the persistence of antibodies in the absence of infection (immunological scar) in older donors at longer risk of infection than younger donors.

## Conclusion

This study presents for the first time the seroprevalence and the evolution of infectious markers in blood donors in a rural area of Gabon. This report showed both an increase in HIV and syphilis distribution, and a reduction in HBV and HCV which is inconsistent with national data and previous studies in urban areas.

### What is known about this topic

- Seroprevalence of blood borne pathogens are well described in urban areas in Gabon;
- Few data are available on blood transfusion-transmitted infections (TTI) in rural areas in Gabon;
- The distribution of TTIs are variable in urban and rural areas in Sub-Saharan Africa.

### What this study adds

- This Trend of blood borne pathogens is the first conducted in blood donors of rural area in Gabon;
- Seroprevalences of TTI were similar in male and female blood donors;
- The seroprevalence of TTI found are lower compared to the seroprevalence described in previous studies in Gabonese blood donors from urban areas.

## Competing interests

The authors declare no competing of interest.

## References

[1] Tagny CT, Owusu-Ofori S, Mbanya D, Deneys V (2010). The blood donor in sub-Saharan Africa: a review. Transfus Med..

[2] Allain JP (2011). Moving on from voluntary non-remunerated donors: who is the best blood donor. Br J Haematol..

[3] Eko Mba JM, Bisseye C, Ntsame Ndong JM, Mombo LE, Bengone C, Mouelet Migolet G (2018). Prevalent hepatitis B surface antigen among first-time blood donors in Gabon. PLoS One.

[4] UNICEF (2013). Annual report 2013-Gabon.

[5] Riou J, Ait Ahmed M, Blake A, Vozlinsky S, Brichler S, Eholié S (2016). Hepatitis C virus seroprevalence in adults in Africa: a systematic review and meta-analysis. Journal of viral hepatitis.

[6] Tonda J, Mickala P, Mombo LE, Etho Mengue JC, Mongo-Délis A, Mbacky K (2017). Séroprévalence du virus de l'immunodéficience humaine, des virus des hépatites B et C et de Treponema pallidum chez les donneurs de sang dans une zone rurale au sud-est Gabon (Koula-Moutou). Journal of Applied Biosciences.

[7] Allain JP, Anokwa M, Casbard A, Owusu-Ofori S, Dennis-Antwi J (2004). Sociology and behaviour of West African blood donors: the impact of religion on human immunodeficiency virus infection. Vox Sang..

[8] Tagny CT, Diarra A, Yahaya R, Hakizimana M, Nguessan A, Mbensa G (2009). Characteristics of blood donors and donated blood in sub-Saharan Francophone Africa. Transfusion.

[9] Tessema B, Yismaw G, Kassu A, Amsalu A, Mulu A, Emmrich F (2010). Seroprevalence of HIV, HBV, HCV and syphilis infections among blood donors at Gondar University Teaching Hospital, Northwest Ethiopia: declining trends over a period of five years. BMC Infect Dis..

[10] Nwankwo E, Mommodu I, Umar I, Musa B, Adeleke S (2012). Seroprevalence of major blood-borne infections among blood donors in Kano, Nigeria. Turk J Med Sci..

[11] Noubiap JJ, Joko WY, Nansseu JR, Tene UG, Siaka C (2013). Sero-epidemiology of human immunodeficiency virus, hepatitis B and C viruses, and syphilis infections among first-time blood donors in Edea, Cameroon. Int J Infect Dis..

[12] Mavenyengwa RT, Mukesi M, Chipare I, Shoombe E (2014). Prevalence of human immunodeficiency virus, syphilis, hepatitis B and C in blood donations in Namibia. BMC Public Health..

[13] Agasa SB, Likwela JL (2014). Barriers to voluntary blood donation in the population of Kisangani in the Democratic Republic of Congo. Pan Afr Med J..

[14] UNICEF (2014). Afrique génération 2030: La démographie enfantine en Afrique..

[15] Mohammed Y, Bekele A (2016). Seroprevalence of transfusion transmitted infection among blood donors at Jijiga blood bank, Eastern Ethiopia: retrospective 4 years study. BMC research notes.

[16] Nagalo MB, Sanou M, Bisseye C, Kaboré MI, Nebie YK, Kienou K (2011). Seroprevalence of human immunodeficiency virus, hepatitis B and C viruses and syphilis among blood donors in Koudougou (Burkina Faso) in 2009. Blood Transfus..

[17] Buseri FI, Muhibi MA, Jeremiah ZA (2009). Sero-epidemiology of transfusion-transmissible infectious diseases among blood donors in Osogbo, south-west Nigeria. Blood Transfus..

[18] Stokx J, Gillet P, De Weggheleire A, Casas EC, Maendaenda R, Beulane AJ (2011). Seroprevalence of transfusion-transmissible infections and evaluation of the pre-donation screening performance at the Provincial Hospital of Tete, Mozambique. BMC Infect Dis..

[19] Xie DD, Li J, Chen JT, Eyi UM, Matesa RA, Obono MM (2015). Seroprevalence of Human Immunodeficiency Virus, Hepatitis B Virus, Hepatitis C Virus, and Treponema pallidum Infections among Blood Donors on Bioko Island, Equatorial Guinea. PLoS One..

[20] Laperche S, Francophone African Group for Research in Blood T (2013). Multinational assessment of blood-borne virus testing and transfusion safety on the African continent. Transfusion.

[21] Orkuma JA, Egesie JO, Banwat EB, Ejele AO, Orkuma JH, Bako IA (2014). HIV screening in blood donors: rapid diagnostic test versus enhanced ELISA. Nigerian journal of medicine: journal of the National Association of Resident Doctors of Nigeria..

[22] Pruett CR, Vermeulen M, Zacharias P, Ingram C, Tayou Tagny C, Bloch EM (2015). The use of rapid diagnostic tests for transfusion infectious screening in Africa: a literature review. Transfus Med Rev..

[23] Eboumbou Moukoko CE, Ngo Sack F, Essangui Same EG, Mbangue M, Lehman LG (2014). HIV, HBV, HCV and T. pallidum infections among blood donors and Transfusion-related complications among recipients at the Laquintinie hospital in Douala, Cameroon. BMC Hematol..

[24] Taye S, Abdulkerim A, Hussen M (2014). Prevalence of hepatitis B and C virus infections among patients with chronic hepatitis at Bereka Medical Center, Southeast Ethiopia: a retrospective study. BMC research notes.

[25] Daw MA, Shabash A, El-Bouzedi A, Dau AA, Association with the Libyan Study Group of H, Hiv (2014). Seroprevalence of HBV, HCV & HIV co-infection and risk factors analysis in Tripoli-Libya. PLoS One.

[26] Mayaki Z, Dardenne N, Kabo R, Moutschen M, Sondag D, Albert A (2013). Seroprevalence of infectious markers among blood donors in Niamey (Niger). Rev Epidemiol Sante Publique.

[27] Rerambiah LK, Rerambiah LE, Bengone C, Djoba Siawaya JF (2014). The risk of transfusion-transmitted viral infections at the Gabonese National Blood Transfusion Centre. Blood Transfus..

[28] WHO (2017). Situation actuelle de la sécurité transfusionnelle et approvisionnement en sang dans la Région africaine de l'OMS-rapport de l'enquête 2013..

[29] Bisseye C, Sanou M, Nagalo BM, Kiba A, Compaoré TR, Tao I (2013). Epidemiology of syphilis in regional blood transfusion centres in Burkina Faso, West Africa. Pan Afr Med J..

[30] Matee MI, Magesa PM, Lyamuya EF (2006). Seroprevalence of human immunodeficiency virus, hepatitis B and C viruses and syphilis infections among blood donors at the Muhimbili National Hospital in Dar es Salaam, Tanzania. BMC Public Health.

[31] Seck M, Dieye B, Gueye YB, Faye BF, Senghor AB, Toure SA (2016). Evaluation of the efficacy of medical screening of blood donors on preventing blood transfusion-transmitted infectious agents. Transfus Clin Biol..

